# COVID-19, science, vaccines and family in a multi origin Latinx population in South Florida

**DOI:** 10.3389/fpubh.2022.997449

**Published:** 2022-09-13

**Authors:** Elena Bastida, Gira J. Ravelo, Pablo Benitez, Jennifer Chavez, Nicholas Metheny, María José Baeza Robba, José Félix Colón-Burgos, Mario De La Rosa, Victoria Behar-Zusman, Olveen Carrasquillo

**Affiliations:** ^1^Department of Health Promotion and Disease Prevention, Robert Stempel College of Public Health and Social Work, Florida International University, Miami, FL, United States; ^2^Center for Research on U.S. Latino HIV/AIDS and Drug Abuse, Robert Stempel College of Public Health and Social Work, Florida International University, Miami, FL, United States; ^3^School of Nursing and Health Studies, University of Miami, Coral Gables, FL, United States; ^4^Escuela de Enfermer, Pontificia Universidad Cat3lica de Chile, Santiago, Chile; ^5^Leonard M. Miller School of Medicine, University of Miami, Miami, FL, United States

**Keywords:** COVID-19, multi Latinx, science, sources of information, vaccines

## Abstract

During the Spring of 2021 in Miami-Dade County, four virtual focus groups were held with 31 participants from four diverse local Latinx communities as part of the Florida Community Engagement Alliance (FL-CEAL) Against COVID-19 Disparities project. The main objective was to explore attitudes about COVID-19 information and prevention strategies among South Florida's diverse Latinx populations, across a broad geographical area. The study used a semi-structured focus group qualitative design and chose participants from four well established Latinx neighborhoods. Participants were mostly women, diversity was strong with birth regions including the Caribbean, North, Central and South America. Though a third (*n* = 11) were born in the United States, almost all (*n* = 28) reported speaking Spanish at home. Three themes and six subthemes were identified to underscore Latinx attitudes toward COVID-19 vaccine uptake or hesitancy. These were: (1) Attitudes regarding vaccine intake; (2) Sources of Information; and (3) Science Education. The degree to which each of these themes exercised influence on vaccine intake or hesitancy varied. The multi origin Latinx participation in the focus groups strengthened findings by broadening representation and discussion. In the end and despite the various national origins, all participants indicated receiving most of their information on COVID-19 related topics from their family, physicians, social networks, and some form of media.

## Introduction

*Yo creo en la ciencia y creo que puede haber la capacidad de desarrollar una vacuna en muy poco tiempo. Sin embargo, han sido tantas las noticias que hemos escuchado, la información que nos bombardean y nos tienen confundidos… pero aún no estoy convencida que sea lo mejor y que esté completamente probado que es lo que debemos hacer. Yo todav*í*a no estoy a punto de convencida de que debemos colocarnos la vacuna* (Angela^1^, focus group participant, Central Miami-Dade County, FL).

I believe in science and believe the means exist to develop a vaccine quickly. However, we've had such an overdose of information that all this bombardment of information has caused us to be confused… this is why I am not convinced that we should get it [vaccine], and that everything has been validated correctly, and that this is what we must do. I am not yet at the point of being convinced that we should get the vaccine (Angela, focus group participant, Central Miami-Dade County, FL).

At the time that Angela expressed the views above, COVID-19 related deaths in the United States (US) had surpassed 483,000 with 27,600,000 confirmed cases of infection (1) and < 2 months had passed since the first COVID-19 vaccine was administered in Florida (2). Yet, despite the development of a groundbreaking vaccine to combat the rising trajectory in both rates and mortality, vaccine hesitancy and fears plagued local communities throughout the US and became strong barriers to vaccine uptake (3). In fact, at the time of this writing, COVID-19 deaths have surpassed one million, and infections rates have reached nearly 89,000,000 cases in the US alone (1). Still, < 67% of individuals residing in the US have been fully vaccinated; this contrasts the higher rates of fully vaccinated individuals in other countries, including the United Arab Emirates (~98%), Portugal (~92%), and Cuba (~87%) (1).

Similar to US national rates, only 68% of Florida residents are reported as having full vaccinations, compared to more than 93% in the District of Columbia (4). In Miami-Dade County (MDC), FL, the peninsula's southernmost county, and site of the current study, Latinxs compose over 69% of the population (5). Compared to non-Latinx White individuals, Latinxs are 200% more likely to die, and 250% more likely to be hospitalized—due to COVID-19 (6). In fact, several studies have shown that the COVID-19 pandemic has disproportionately impacted marginalized minority communities (7–9) and Latinx communities in MDC are no exception.

During the Spring of 2021 in MDC, four virtual focus groups were held with 31 participants from four diverse Latinx communities as part of the Florida Community Engagement Alliance (FL-CEAL) Against COVID-19 Disparities project (10). The groups were conducted to explore attitudes about COVID-19 information and prevention strategies among South Florida's diverse Latinx populations.

At the start of the FL-CEAL project, the only CDC recommended prevention approaches based on research consisted of mitigation strategies such as social distancing, wearing masks, washing hands regularly, and isolation when or if infected (11). During this time, much was still unknown about the epidemiology of COVID-19; hence, misinformation and fearmongering quickly became widespread across social and mass media outlets regarding the origins of COVID-19, its effects, infection rates, and mortality (3). Simultaneously, scientists were working to understand the new virus, while health care providers risked their personal health and safety in overwhelmed and understaffed hospitals (12). Concurrently, the US government became divided on how to address the pandemic, causing division among citizens as well (3). On December 11, 2020, the United States Food and Drug Administration (FDA) issued an Emergency Use Authorization (EUA) for the first COVID-19 vaccine (Pfizer-BioNTech) (13). One week later, the FDA issued another EUA for a second vaccine (Moderna) (13). Two months after the second vaccine was issued, the FDA issued the third EUA (Janssen) (13); full FDA approval for COVID-19 vaccines were the fastest in FDA history (14), but sparked widespread misinformation, misconceptions, and conspiracy theories about the vaccine itself (3).

It was in this highly politicized context regarding the COVID-19 pandemic and vaccine that we held focus groups and community observations in MDC, Florida, in early Spring 2021. The purpose was to explore how diverse Latinx residents—of various national origins, socio-economic status, political beliefs, and sexual orientation—viewed the COVID-19 pandemic and recently developed vaccines, while also identifying potential barriers or motivators to receiving the vaccine and promoting it to family members and friends.

### Design

The current study used a semi-structured focus group qualitative design. Four virtual focus groups (*N* = 31 overall) were conducted throughout Miami-Dade County (MDC) from January 21 to February 15, 2021. Participants were recruited from high density, multi-origin Latinx communities—specifically, North Miami-Dade County (North-MDC) (*N* = 8), Central Miami-Dade County (Central-MDC) (*N* = 8), South Miami-Dade County (South-MDC) (*N* = 8), and an additional county-wide sexual gender minority group (SGM) (*N* = 7). Participants were recruited by an outreach worker and are active community residents with no formal roles in the agencies that facilitated their recruitment. Outreach protocols established by (FL-CEAL) and the Center for Latino Health Research Opportunities (CLaRO) (a Florida International University and University of Miami collaborative research center) were instrumental in obtaining community support to remotely contact and recruit participants. Specifically, the study utilized the protocols directing community health workers to target minority communities with outreach focused on education and information regarding COVID-19 research and prevention efforts. The design leveraged South Florida's multi-origin Latinx population to conduct outreach and recruit diverse Latinx participants.

## Methods

### Community selection and recruitment

Recruitment began on January 10, 2021, and was conducted in collaboration with various social service agencies strategically located throughout MDC, FL. A Latinx community outreach worker contacted local agencies to explain the research study and participation criteria. In designing the recruitment plan, agencies were selected on the basis of their Latinx client volume and their commitment to community participatory research with our academic institutions.

To obtain broad geographical inclusion, participants were chosen from four Latinx communities within MDC. These included: (1) North-MDC where Latinx populations compose 29.5% of the population; (2) Central-MDC, including the neighborhoods of Allapattah, composed of over 76% Latinxs, predominantly of Dominican Republic origin and Little Havana, composed of 95% Latinxs, historically of Cuban descent; however, in recent years the area has become more diverse and home to immigrants of Mexico, Central America, South America, and the Caribbean (15, 16). Finally, (3) South-MDC centered on the town of Homestead, predominantly a farm working community, which is composed of 68% Latinxs with higher rates of foreign-born residents (36%) compared to the rest of Florida (20.7%) (17). Given the study's inclusion goal, a fourth focus group was conducted to include representation from the large Latinx sexual and gender minority (SGM) population in MDC. Purposeful sampling, widely used in qualitative research, was used to identify, and select the most information-rich individuals, this approach was particularly helpful when working with limited resources and time, suggested by Patton (18, 19). Sampling recruitment procedures yielded a study population inclusive of the broad Latinx community within MDC, that was knowledgeable about the respective targeted communities. Participants in the four focus groups included active community members, expected to relate broad perspectives, opinions, and concerns regarding the COVID-19 vaccine in their respective communities and, specifically, their personal, family, friends, providers and neighbors' attitudes and behaviors regarding the newly developed vaccine.

### Participants

For all focus groups, the following inclusion criteria were used: being (i) an adult aged 18 years or older, who is (ii) a leader or active member of the target community that (iii) consented to complete a one-on-one pre- and post-survey and (iv) consented to attend a 90-min Zoom focus group and was (v) able to understand and effectively communicate in English or Spanish. Although measures were taken to ensure a diverse range of ages and genders, participants in the three community focus groups consisted of nearly 88% females for all three geographic groups (North-MDC, Central-MDC, South-MDC), while the sexual and gender minority group (SGM) consisted of six males and one transgender female (See Tables 1, 2). Data generated from these groups contributed an additional and diverse perspective on COVID-19 vaccine knowledge and opinions, given their distinct national origins, diverse experiences, and past and current involvements in their respective communities. It is noteworthy that one member of the SGM group remarked: “this is not the first epidemic my community has experienced.”

**Table 1:** Characteristics of participants.

	***n***	**%**
**Gender**		
Female	21	70.0
Male	8	25.8
Transgender	1	6.7
**Language spoken at home**		
English	2	6.7
Spanish	28	93.3
**Region of birth**		
Caribbean	4	13.3
Central America	10	33.3
North America	12	40.0
South America	4	13.3
**Race**		
Black or African-American	2	6.7
White	19	63.3
White, American Indian or Alaska Native (Biracial)	1	3.3
White, Black or African-American (Biracial)	1	3.3
Prefer not to answer	7	23.3
**Household income**		
Less than $15,000	2	6.7
$15,000–$19,999	1	3.3
$20,000–$24,999	2	6.7
$25,000–$34,999	4	13.3
$35,000–$49,999	7	23.3
$50,000–$74,999	11	36.7
$100,000 and above	3	10.0
**Education**		
Some high school	1	3.3
High school graduate or GED	3	10.0
Associate's or technical degree	8	26.7
Bachelor's degree	15	50.0
Graduate degree	3	10.0
**Age (in years)** ***M*** **= 44.4**, ***SD*** **= 13.7**		
20–29	5	16.7
30–39	8	26.7
40–49	5	16.7
50–59	8	26.7
60–69	4	13.3

*Median age* = *40.5*.

**Table 2:** Individual characteristics.

**Fictitious name**	**Education**	**Age**	**Gender**	**Country of origin**	**Household income (1,000 s)**
**South Miami-Dade County (SMDC)**					
Elena	BA	52	Female	Colombia	15–25
Antonina	BA	58	Female	Mexico	25–35
Maria	MA	41	Female	United States	50–75
Victoria	HS	34	Female	Nicaragua	100 and above
Angela	BA	59	Female	Colombia	50–75
Sofia	AA	60	Female	Colombia	25–35
Rosa	HS	47	Female	Panama	50–75
Diana	AA	62	Female	Nicaragua	15–25
**Central Miami-Dade County (CMDC)**					
Martina	AA	30	Female	United States	25–35
Mercedes	AA	58	Female	Guatemala	35–50
Gabriela	BA	49	Female	Nicaragua	50–75
Julieta	BA	61	Female	Nicaragua	35–50
Carlos	BA	65	Male	Honduras	15–25
Nina	BA	40	Female	Nicaragua	100 and above
Natalia	BA	55	Female	Nicaragua	25–35
Raul	AA	22	Male	United States	35–50
**North Miami-Dade County (NMDC)**					
Luis	HS	40	Male	Argentina	50–75
Elsa	HS	39	Female	Cuba	50–75
Barbara	MA	29	Female	United States	50–75
Ana Maria	MA	56	Female	Cuba	35–50
Rebeca	BA	37	Female	Cuba	35–50
Teresa	AA	20	Female	United States	50–75
Carmen	BA	37	Female	Dominican Republic	35–50
Cecilia	BA	38	Female	United States	35–50
**Sexual Gender Minority (SGM)**					
Camille	AA	57	Transgender	USA	<15
Jesus	BA	33	Male	USA	50–75
Fernando	AA	26	Male	Honduras	50–75
Jorge	BA	35	Male	USA	100 and above
Miguel	SHS	-	-	-	-
Daniel	HS	67	Male	USA	50–75
Christian	BA	26	Male	USA	50–75

*N* = 31.

### Data collection

Focus groups were chosen as the primary mode of data collection to gain insight and explore knowledge, perceptions, and opinions regarding COVID-19 and rejection or acceptance of the vaccine. Focus groups were conducted virtually (*via* the Zoom online conferencing platform) during separate days for each neighborhood and the SGM group. All focus groups were held within a 3-week period to limit participants' exposure to media messages and frequently changing sources of information. Groups were conducted in the early evening to accommodate participants' schedules and help facilitate participation. Before each focus group started, participants completed a short demographic survey. Each ~90-min focus group was facilitated by a study co-investigator and assisted by a co-facilitator. Whenever clarity was needed during the course of the focus group, the moderator rephrased the question or asked from a different point of view, as suggested by Krueger and Casey (20).

The community focus groups were conducted primarily in Spanish and facilitated by the same team and using the same focus group guide to maintain contextual consistency. The SGM was the only bilingual (English and Spanish) group in which both languages were used, as needed. While developing the focus group guide and preparing for the focus groups, the first author watched local Spanish language daily television news to gain insight into the community's exposure to the COVID-19 related information provided by these outlets. Concurrently and in an effort to contextualize place, fieldwork observations were conducted at retail pharmacies and food markets located in targeted neighborhoods.

As indicated in this paper's opening quotation—and supported by our research team's fieldwork observations—confusion about the development of the vaccine and its possible deleterious effects was extensive in these communities at the time focus groups were conducted. Spanish media focusing on COVID-19 and vaccine-related information, varied according to the different audiences to which it reached. For example, most radio programs in Spanish were guided by countries of origins, political orientations, and most frequently, religious affiliations. The media's influence on the Latinx community's response to the vaccine was notable at the time focus groups were held because local media broadcasts transmitted widely different rumors and stories. For example, popular during late Fall 2020 and early 2021—immediately preceding and concurrent with our focus groups—were media stories about a pastor at one of the largest Spanish language mega-churches in MDC who discouraged uptake of the vaccine and instead urged “taking believe in divine immunity” (21). Media and field observations were instrumental in developing focus group questions and probes.

### Data analysis

Focus groups were recorded using Zoom platform and collected audio/visual recordings were watched independently by the first and second authors, each doing a line-by-line analysis to identify major themes. Whenever questions emerged, one additional author was asked to review parts of completed recordings for further discussion and clarification. Transcript-based analysis was employed, following Krueger and Casey (20). Transcripts were submitted to four comprehensive reviews using original recordings and field notes. Transcripts from the three community focus groups were first discussed and analyzed between the first two authors, who were present at all community focus groups. The audio/video recording of the SGM focus group was watched and reviewed for topics and consistency five times by the first author, followed by five additional views and reviews, conducted jointly by the first, second and third authors, including a careful line-by-line comparison between the transcript and the actual recording.

Data analysis was performed in the language participants used with Spanish being the prominent language; therefore, the line-by-line analysis was performed in Spanish to avoid a third level of data interpretation. Translations were performed only for the purpose of reporting results. Several constructs from Social Cognitive Theory (22) primarily, self-efficacy, outcome expectancies, reinforcement, and behavioral capability guided the data collection and analysis.

## Results

Major theme and subtheme selection came about as an iterative and collaborative process among the authors. The first and second authors independently read each of the transcripts and identified the themes presented in each focus group. Once themes were identified for all four focus groups, the first two authors and a third reader who had not coded the transcripts identified the salient themes and agreed on their overarching structure. The study authors met to review the transcripts and selected themes and agreed on the findings.

### Major themes and subthemes

#### Theme 1: Attitudes regarding vaccine intake

Analysis of data from late 2021 suggest that participants' attitudes toward the vaccine were similar among the four communities of Miami-Dade County. Participants in all groups offered similar arguments to explain and support their hesitancy toward COVID-19 vaccination, but each group included individuals who trusted the vaccine and did so for similar reasons.

##### Subtheme 1: “A wait and see”

The most frequent theme regarding COVID-19 vaccine hesitancy, mentioned on 32 different occasions by participants, was a “wait-and-see” approach, mainly stemming from beliefs that the vaccine “was developed too quickly.” For example, one woman participant expressed her concern over the development timeline, by stating:

…when checking the Internet, in previous years, how long does it take for a vaccine to hit the market? Often, two, three, even four, or five years, and this one [was done] in a matter of months... when “they” (here assumed scientists) still don't even understand well what Coronavirus is. (Carmen, NMDC)

In the above comments, Carmen shares knowledge about vaccine development, which informs her behavioral capability and subsequent exercise of self-efficacy when deciding on whether to get vaccinated or not. Alternatively, others expressed hesitancy because of fears of side effects. Amelia from Central-MDC suggested: “I think it's best to maintain good health and wait… to see what's going to happen to those people with horrible side effects.” Similarly, one participant from the South-MDC farm workers focus group reminded fellow participants that all medications have side effects and continued by noting, “If it [the vaccine] affects other organs, it may protect you, but it's affecting something else. So, I think we have to wait a little longer to see what the effects of the vaccine are.”

Vaccine hesitancy was common even among participants who expressed support of science. One such participant, as presented in the opening quotation of this paper, expressed reluctance resulting from overwhelming media information:

…so, the more news you listen to, the more questions arise with less answers. I believe in science; I believe in the companies that develop it [vaccine], and I believe it can be a good thing, but I'm still not convinced that it's the best thing for us at this time, and that it has been completely proven that it's what we should do… I'm still not at the point where I'm convinced that we should get the vaccine. (CMDC)

Similar to the participant above, Jesus, a participant from the SGM group indicated: “What you most hear in the community is the uncertainty, or that, ‘I don't know what can happen, rather than it's good or bad.”

Those who expressed hesitancy unanimously expressed their concern for the speed at which the vaccine was developed—similar to findings reported from a recent study by Bateman et al. (23). Though levels of mistrust were high at the time the focus groups were conducted, it was generally related to vaccine quality and effectiveness. Others who manifested mistrust for the vaccine also indicated a wider skepticism for the structure and practice of medicine in the US, suggesting wariness of medicine in the US as a for-profit business, as expressed by a North-MDC participant and agreed by most participants present at that focus groups.

Participants' statements frequently revealed fears rooted in past events and confusion by what they considered overwhelming daily information from multiple sources, such as television news coverage, radio talk shows, internet and social media, and word of mouth. Fernando, a participant from the SGM group, indicated that media messaging surrounding the vaccine “lacked transparency and fueled issues of mistrust and hesitancy.”

…*I didn't look up more details, but I remember... the Florida doctor, that he took the vaccine, and then 16 days later, he died. And then, in the news, the wife said that ‘Oh. It's due to the vaccine.' And Pfizer … said, ‘No. It has nothing to do with the vaccine.' But then... the CDC is investigating the case. So, what I don't like is how the media will blast the whole article... kind of to put fear into the people about the vaccine*.

##### Subtheme 2: “Sometime in the future, not saying never”

Vaccine hesitancy and vaccine acceptance are not mutually exclusive and participants comments reflect both. Indeed, this was the case for Fernando. In response to the facilitator's probe on whether he perceived any personal consequences to receiving the vaccine, he responded: “Oh, yeah, I have no cons against it, for sure. When it is available for me, I'm the first person to go.” Unlike the above participant, others admitted they were “not ready” or “still searching” to receive the vaccine. However, they were not opposed to it, indicating their willingness to receive it at a later time when the vaccine had been more adequately tested, or when more was known about its side effects. Some made exceptions, however, to their “wait-and-see” opinion. One participant in the SMDC group, who was told that it could be required to travel to a foreign country, almost immediately modified her earlier “wait-and-see” approach to the vaccine and expressed that she would get the vaccine within “a moment's notice,” if required to travel abroad to visit her loved ones. A woman of Central American origin from the same group added, “if for international travel the vaccine is mandated, then I will get it immediately to visit my family.” Others who had expressed hesitancy earlier agreed to vaccination, if necessary to travel, almost immediately modifying their earlier position resulting from the recently received information.

Among those who said that they would get vaccinated were several participants in the SGM group who acknowledged that their motivations were related to their comorbidities, which increased their risk and fear of infecting others. These included references to living with an older family member, attending a social event, being a “vector” of the virus to others or a desire to return to “normal”.

Additionally, one participant in the SGM focus group, Camille, drew a parallel between the COVID-19 pandemic and the experience of the SGM community with the HIV epidemic. She ended her comment by advising others to get the vaccine:

I want to say I came through another epidemic a long, long, long time ago. So, yes, there was a lot of stigmas, probably just as much stigma as is going on with the pandemic today, but I educated not only me, but I educated other people, and that helped diffuse the fear a lot. I've seen that epidemic progress. I'm talking about way from ‘80s, I've seen the epidemic progress. So, when it comes to this pandemic, I can't emphasize how important it is to educate them people. Me, personally, if they're in a category that are a lot of—I think I heard people say underlying conditions—that they need to be vaccinated, my advice would be to get it.

Moreover, while there were many who expressed hesitancy, of the 31 focus group participants, only three gave an adamant “never.” Interestingly, among the three who expressed the most hesitancy, two, soon after, received the first dose and shared the news with the outreach coordinator. Despite earlier discussions on hesitancy, mistrust, and other concerns, over one-third of participants expressed willingness to get vaccinated “as soon as possible”—not finding it necessary to wait for additional information on the vaccine and indicating strong efficacy. A woman from South-MDC expressed satisfaction about receiving the vaccine as early as mid-January 2021, during a time when the majority of the US population had not been vaccinated. She said:

Well, I called my primary physician, I also have several other specialists, since I have a chronic health condition that requires frequent medical visits, and my physician told me that his practice was recommending intake of the vaccine. I went ahead and got it. A day later, I heard my daughter say, “the person I most wanted to get vaccinated was my mother, and she already got it.” When I heard her say this, I felt so much better. I already have my appointment for my second shot.

The above woman shared that her daughter, a nurse, had been heavily involved with COVID-19 patients and at “the center of the crisis.” Another participant from the same community indicated that she would get vaccinated as soon as she could make an appointment, even when questions remained about possible side effects. Still, referring to a well-known Spanish proverb, she shared, “it is always easier to prevent rather than to treat.”

In general, most participants who expressed hesitancy also observed that it was not whether they will get it or not, but rather when they would feel or think “sufficiently secured getting it.” Only three participants appeared reticent at the time, and one said that “it would take a lot of information [from those she most trusted] for me to be ready for the vaccine,” illustrating constructs, such as expectations, expectancies, and expanding her behavioral capability. While some ignored the negative comments and were ready to be vaccinated, as a participant from Central-MDC (Little Havana) said, “the sooner the better.” Others, who expressed hesitancy in terms of a “wait-and-see” attitude, also acknowledged the possibility of getting “the vaccine sometime in the future.” When carefully analyzing the data line-by-line, two groups were identified: one group who said they were ready for the vaccine, and a second group that professed a “wait-and-see” attitude, with most noting that, “not getting it now, does not mean we will never get it.” This second group expressed dissatisfaction with the information being received at the time from mass media, indicating that “at a future time when more scientific information became available and when the numbers of the vaccinated increased, they were more likely to get it.”

#### Theme 2: The media and other sources of information

##### Subtheme 1: “We have a cocktail of information”

Beginning with the first focus group, sources of information emerged as a significant theme in participants' narratives, and several participants found the source to be of utmost importance when making decisions about COVID-19 exposure, mitigation practices, or vaccination. A participant from the N-MDC focus group referred to the CDC as a “most trusted source of information on COVID and the vaccine.” Data analysis revealed that participants from all targeted MDC communities unanimously trusted information from their physicians, immediate family, or both. Those who gave higher priorities to trust in their physicians, were those who also indicated having chronic conditions that required long term relationships with their physicians. Fernando, a participant in the SGM group made a particularly strong reference to his physician as the person most trusted when stating: “I suffer from chronic conditions, diabetes, asthma, among others… I trust my physician.” Similarly, a woman with chronic conditions from South-MDC indicated that she had consulted with her physicians and, following their advice, she elected to be vaccinated. Others, not reporting major chronic conditions, were more likely to indicate close family members as their most trusted sources, especially when the relative was in the health field. Ranked close to their physicians and family members, were other health professionals (such as pharmacists), and reputable websites (e.g., American Medical Association) and university websites (e.g., the Cornell University website was mentioned directly by Rebeca, a North-MDC participant). Over half of all community participants indicated that reputable and university websites were also important resources; the latter was frequently mentioned (5+ times) during focus group sessions when participants sought to justify their various positions by citing sources of information and informants beyond their close friends and family members.

More distant, yet important, sources of information frequently reported by participants were media personalities (e.g., television hosts) and recognized television networks, such as US CNN, FOX News, and local and Spanish language broadcasting networks. For example, Ana Maria, a participant from North-MDC, whose daughter lives in Spain, was adamant in her opinion that Spain's major national television network, TVE (Television Española), was her preferred source of COVID-19 media coverage. Ana Maria noted, “I watch TVE regularly and their hosts are very clear when speaking about COVID.” When asked why she prefers TVE for COVID-19 news and information, rather than US networks, she noted, “they are more trustworthy, they provide clarity to the topic, their presenters stay away from politics; actually, they refer to science not politics when conveying news and mitigation practices.”

Participants were also frequent users of online sources of information, among which YouTube was the most frequently cited. Angela from Central-MDC observed:

Well, I have membership in various channels, and in a YouTube channel, there is a host called Gary Burg who is a medical doctor and also very young, he is fabulous because he explains all illnesses very detailed, and he tells us what needs to be done in order to eat better, live better, the connection between mind and body, how to sustain stable health. I also watch Dr. Hyman who has a clinic in New York... [and] there are lots of good medical doctors on television that present programs that help with health that are free and these are the programs that I follow and help me in making my health decisions.

Within the broad topic of media as a source of COVID-19 information, participants in the North-MDC focus group observed that US media messages appeared contradictory and confusing. When asked, “What comes to mind when you listen to COVID-19 news, read about COVID-19 (online, newspapers), or talk about it to your friends and family?” Ana Maria, a Central-MDC participant noted: “Listening to contradictory “things” (*cosas* in Spanish). Here, “things” (*cosas*) was a word frequently mentioned during focus groups when participants were referring to the information they heard and watched on COVID-19 news. When further probed about the meaning of “things,” there was hesitation from Ana Maria and those in the group who agreed with her; participants then explained that, when referring to news broadcasts, “things” indicated noise, such as information not to be taken seriously or trusted, whether from television, radio, or print. When referring to “things,” Ana Maria clarified, “I speak about mistrust in terms of local media coverage of COVID news and information, I am cynical about all the “things” that I listen on a daily basis.” She adds:

My personal opinion is that health, in this country, is a business; that is how I see it. It is all about profit and COVID is all about inducing panic. [She continues], so when I say all those “things", I mean all the irrelevant and redundant comments that people bring up that lead to panic, or denial. I say that management of COVID information has been very poor. I have had multiple negative experiences.

Similarly, but from a different ideological position, Carlos from Central-MDC indicated:

We have been manipulated for a long time by 24-hour news and now with COVID, one media source says, “one thing,” while the other says another, an expert offers an explanation and another says the opposite, the media promoted a situation of uncertainty and doubtfulness. Media channels and the government need to monitor what is said about COVID and the information that constantly circulates. Otherwise, we get the mess we are in.

An older woman from Central-MDC, Nina, responded by noting:

I am very much in agreement. I believe that we have a cocktail of information, lots of very bad information. “Things” are said without proof or verification, creating fear in society. I believe every person has the capacity to decide.

Diana, from the South-MDC farmworkers community, further adds to this theme by suggesting:

In order to trust the vaccine, the media need to be exact and precise in circulating news about the vaccine. They should not lie, not say this or that, but to be precise in their messages, not to say one thing one day, and the next reverse it. For me this is very important because most of us are constantly watching the news, I watch news all day and rely on what I hear. I have heard that many are dead in other countries, in Germany and Norway, I don't remember all the places, because they received the vaccine, so that makes me think twice on when I should get it. Perhaps wait longer to make sure.

Participants from the SGM group also expressed dissatisfaction with media coverage of the vaccine, and the influence that it has on individuals who rely on it as a source of information. Specifically, Christian said:

I do feel like a lot of the dividing opinions have also come of where people choose to get their news from misinformation just for the sake of selling clicks and selling advertisements. And as long as it makes a good headline, they'll post whatever. I also think a lot of that of looking around in Facebook and, now online, you have the option to cherry pick whichever news you want […] I do feel like the media has created a lot of confusion.

##### Subtheme 2: “My biggest fear is my family, my grandmother.” Information and support across generations

Comments pointing to family and relatives among the most trusted sources of information were common among all focus group participants. These were reinforced when the children of participants, or other relatives, worked in the health field. Participants who reported being vaccinated, or scheduled to be vaccinated, alluded to recommendations received from their physicians and/or their children or close relative in health fields. In fact, children, particularly those in the health field were the ones with the most influence on decisions to vaccinate, followed by physicians. Additionally, trusted dyads, such as mothers and daughters, were particularly important in all instances where the parent's health was concerned. It was notable that references to children, or close relatives, in the health field were heard in all community focus groups.

The current Information and Support Across Generations theme was also evidenced by a mother from South-MDC who indicated that she had been vaccinated, following advice she received from her adult daughter, a nurse. Data from all focus groups support findings suggesting that younger family members are more likely to keep up with broader cultural messaging than older family members. Such was the situation discussed by younger participants of the SGM group, born in the US (five out of seven), who indicated that they played a role in informing, advising, and sharing a sense of responsibility for older family members. For example, Fernando notes:

My biggest fear especially with my family was right before Thanksgiving when my partner got COVID 19, and I feared for my grandmother. So luckily, she was not living with me, but if she needs my help, or anything, I try to limit my exposure to her. So, she got the vaccine now…. then obviously when I get it (the vaccine) it eliminates the fear of like Oh, I can potentially give it to her.

Fernando, further indicated: “For example, my grandmother, the second I heard about the elderly people getting the vaccine, I already had her phone number, I was willing to do the appointment for her, but she had an appointment already.”

Another participant from the SGM group, Jesus, also noted that when asked about the vaccine by his mother-in-law, his suggestion was: “…go for it. …that was my suggestion because that happened to be my mother-in-law.” The above comments indicate that this younger group, plays the role of informers and advisors partaking in culture and guiding their older family members.

#### Theme 3: Science and education

##### Subtheme 1: “We need to keep super informed:” Trust in science

Participants' views on science, scientific information and scientists emerged as a third major theme in focus groups discussions. At the time focus groups were conducted, while the pandemic was at its height, most participants, across all focus groups, expressed trusting views of science and scientists. References to scientific information, knowledge, and scientists were common throughout all focus groups. As in the opening quotation, most participants acknowledged respect for science, frequently referring to the CDC website and to “Dr. Fauci,” who most, if not all, knew from the various news outlets they watched.

Upon concluding a second line-by-line review of focus group data, no refutations of science were found in the transcripts; even when expressing hesitancy about the vaccine, no participant openly or directly expressed a disbelief in science or scientific methods. When vaccine hesitancy was expressed, it was in relation to doubts about the methods of science not being appropriately or sufficiently applied during development and testing. For example, a participant from South-MDC noted: “I keep reiterating to friends and family that we need to be super informed of the latest scientific information, this virus is all over the world; in the months ahead, we will learn a lot more about it.”

When referring to scientists, a woman also from South-MDC, Victoria, complained by saying:

The problem is, they first said it was an unknown virus… then suddenly they make a vaccine. How can they make a vaccine for a virus they don't know? …in other words, so much has been said that not even they [scientists] really understand the virus, yet they already have a vaccine for a virus they don't understand? …I will not be convinced.

In response, participant Maria added, “When she travels, then she'll have to take it,” but Victoria replied with, “Not even, I'm willing to not travel...”

When asked, when was “the right moment to get the vaccine,” Maria from South-MDC, responded:

When scientists produce a medical journal that says: “People who were vaccinated have become immunologically protected, they are now immune to the virus”; because I want to know, who are these people participating in vaccine trials? Is it a Puerto Rican, a Colombian? A US born Colombian or a foreign-born Colombian? A Puerto Rican like myself? born there… or here? You know what I mean? So… when I hear that they did trials with a group like *me*, then I'll get it (vaccine).

Jesus from the SGM focus group shared his trust in science, noting:

What you mostly hear in the community is the uncertainty, or that “I don't know what can happen,” … But it seems like everything is running fine and I trust science. There was research before, so that's it, Thank you.

Rebeca from North-MDC repeatedly said that she never watches the news; however, she followed, “… what they [scientists] say especially in scientific journals which are peer reviewed. If I find the articles interesting, I read it; if not, I pass.”

Finally, only three participants made references to alternative health choices: two participants described the YouTube doctors they watch regularly, who endorse a holistic curative approach to illness through nutrition; the third participant, a woman, shared her observations about an Asian couple who used herbal teas to treat COVID-19. Others in all groups demonstrated strong support and respect for science, scientists, and health professionals, despite reservations about the vaccine rollout.

##### Subtheme 2: “Getting Educated.” Education, studying, and following mitigation recommendations

Participants felt strongly about education, and phrases such as “getting educated” and “becoming educated” were expressed frequently during focus group discussions, especially when referring to following CDC recommended mitigation practices or choosing to ignore them.

Angela from the South-MDC group made a case for education.

I want to share with the group that above everything else there is education [Pause]. I think that the community needs to be educated on a daily basis and addressed with a great deal of sensibility. I believe that first we need education to make people aware that despite whatever our needs, we should not be out shopping without protection, especially those with COVID symptoms should not go to public spaces without being tested first, that, when necessary, they follow mitigation practices.

A respondent from the same group agreed:

I think that education is a fundamental part. We must strengthen education programs in our communities, make people more aware, conscientize the community, so they engage in mitigation behaviors.

Rosa added:

I believe that as time goes by, we will see this illness developing and further infecting others in large scale. This is the time when we are going to have to run and gain confidence on the vaccine and learn how to educate families, like mine.

Cecilia from North-MDC observed, “people need to make educated decisions, to share information.” Alicia, also from North-MDC, followed: “people need to read, share information, search databases, so they become more educated.” Susana agreed: “studying is important, community leaders should educate the community and provide information.”

Participants in the SGM focus group also felt strongly about education. Camille emphasized the need for education when speaking on the vaccine, noting: “education is the key to a lot of things. Find out as much as you can about the vaccine, and don't go at it just because it's fear of something.” Similarly, another participant—when referring to the absence of mitigation practices among sex workers—commented: “…so there might be another way to allow them to access education, or to access the workforce, education, to get more dignifying degrees, then there is a greater sense of worth.” Another participant, also from the SGM group, added that “education decreases the stigma associated with partner violence in our communities.”

## Discussion

Three themes and six subthemes were identified to underscore Latinx attitudes toward COVID-19 vaccine uptake or hesitancy. The three major themes that emerged were: (1) Attitudes Regarding Vaccine Intake, with two subthemes: (a) vaccine hesitancy and (b) vaccine acceptance; (2) Sources of Information, with two subthemes, (a) the media and other sources of public information, and (b) information and support across generations; and (3) Science and Education, with two subthemes: (a) trust in science and (b) education, studying, and following mitigation recommendations. The degree to which each of these themes exercised influence on vaccine intake or hesitancy varied.

Data analysis from the four focus groups provided the opportunity to reach, identify, and report on multi-origin Latinx participants' attitudes toward the vaccine, including themes on science and medicine while also highlighting reasons for vaccine hesitancy. We expect findings from these groups to assist in establishing the foundation for an improved and wider understanding of Latinx vaccine behaviors in general and their openness to vaccination.

Within the theme of “valued sources of information,” participants from all focus groups viewed their physicians, immediate family, or both as their major and trusted source of vaccine information and inclination. Physicians and families, whether nuclear or semi extended, or both, were their major and most frequently solicited source of information on all aspects of the vaccine, including not only recommendations or rejections, but also information and discussion on the scientific merits of the vaccine and/or getting vaccinated. Participants expressed less engagement with neighbors or distant family members, even when in close neighborhood proximity.

Findings suggest that participants held strong views on science. They were often eager to find evidence in support or rejection of vaccine intake based on their access to the readily available scientific information. Those participants frequently searched through online sources or local media. All participants were familiar with using virtual modes of communication and demonstrated familiarity and reliance on websites that promoted different vaccines perspectives.

Findings presented here have to be interpreted within the multi-origin, broadly diverse Latinx population of Miami Dade County, not only diverse in national origin but also in socio-economic characteristics. However, above findings provide a preliminary outline of the groups' attitudes and behaviors on the vaccine, as well as their views on science and respected sources of information. Participants acknowledged respect for science, professional expertise, and information. However, vertical networks of family members in health professions and horizontal networks of friends and neighbors were also important sources of information. Vertical family networks were valued sources of information and support. Preferably, advice and information were sought from family members who were health professionals (e.g., nurses, pharmacists, physician assistants or other health professionals, including physicians). Some participants in the SGM group who acknowledged close proximity to physicians and other health workers, many in second generation vertical family dyads, advised and encouraged other family members, most often a grandmother or mother-in-law, to become vaccinated. Current findings are similar to studies that have shown Latinxs are likely to get their health information from sources such as physicians, family, friends, and social networks, and some form of media (24). In a more recent study, the Pew Research Center reported that although Latinxs have used radio and newspapers as sources of information historically, television and the Internet are more widely used now. In fact, the Pew study also found that, among Latinx individuals, there were significant declines in use of radio and newspapers as news sources between 2006 and 2016; even television, as a source of information, declined during that same period (25).

In contrast, between 2006 and 2016, there was a 37% increase (74%) of Latinxs reported use of the Internet (including social media and smartphones), of which 66% of those used it to search for health information. Moreover, 41% reported their decisions to treat health conditions were influenced by what they saw in media (25). As such, these studies suggest that the internet is increasingly becoming a main source of information for Latinxs, as is also suggested from the current research findings.

Finally, fear-of-deportation due to undocumented status, though recently reported by Bateman et al. (23) as a hurdle to vaccine uptake among Latinx individuals in Jefferson County, Alabama, did not emerge as a theme in any of the four focus groups with multi origin Latinx populations in MDC. We explain the absence of this topic in our focus group discussions by noting that a third of participants were US born (*n* = 10) and obviously not affected. Second, the demographics of Miami-Dade County (MDC), where 54% of the population is foreign born (5), provide a positive receiving context for all Latinx immigrant populations which we suggest account for the absence of the fear-of-deportation topic among those who were foreign born. However, agency involvement, particularly when encouraging participants to volunteer for the focus group, could have resulted in that those most community active were probably more likely to have formalized their immigration status.

## Strengths and limitations

We find the multi-origin Latinx representation among focus group participants in Miami-Dade County strengthens the findings presented here. Results contribute to the literature on the Latinx perspective on the COVID-19 pandemic and consequent vaccine attitudes that cut across Latinx groups from diverse national origins. Multi origin Latinx populations are increasingly becoming part of the demographic profile of the largest metropolitan counties in the US and thus important for research. Focus group topics aimed to identify shared factors underlying participants' reasons for accepting or rejecting the COVID-19 vaccine while promoting an open environment for participants to share their hesitancy or their vaccine acceptance.

By delving into participants' reasons for vaccine hesitancy or acceptance in a multi-origin Latinx population, findings yielded by this study can be useful in designing health promotion and prevention initiatives that address COVID-19 related fears among these subgroups. Results may also be extended beyond the targeted aims to include different health related issues and concerns. Outreach messaging to these communities should be anchored by scientific support, the authenticity of the messenger, or preferably both. Focus group data presented here suggest that vaccine messaging and endorsements are best received when coming from a trained health professional or a grown child or close relative, especially when the latter are trained in the health professions. Study findings have the potential to contribute to designing interventions aimed at multi-origin Latinx groups. To that end, findings from this study guided the development of a short intervention where tailored COVID-19 public health messages, sourced from National Institutes of Health and Centers for Disease Control and Prevention, were sent to focus group participants *via* WhatsApp. We find that focus groups results from the multi-origin Latinx presence in this study facilitated a broad outreach to diverse Latinx origin groups in Miami Dade County.

Furthermore, the research team strove to promote an open environment for participants to share their hesitancy and their acceptance. Notwithstanding the strengths, this study had several limitations in the recruitment of the sample through well-established community agencies. Though fostering an inviting environment where they expressed their opinions openly, agencies may have selected participants and/or promoted the study to those community members who displayed involvement and support of their programs and activities. Hence, participants may have been more engaged, more educated, and more likely to express opinions than perhaps members of the same communities less acquainted with these agencies, or less likely to be community leaders. Despite these limitations, the results presented here contribute to the literature on vaccines in general and present a broader perspective on vaccine attitudes in multi-origin Latinx populations.

Findings and results presented here on multi origin Latinx groups in South Florida while strongly supportive of findings obtained with more homogeneous Latinx groups (26, 27) such as those centering around misinformation and distrust of health information sources also differed in that insecurity in the form of fears of loss of employment and deportation were not salient among our multi origin Latinx participants. On the other hand, themes of family-related stress from changes in the home dynamic due to increased utilization of shared space and concerns of social isolation due to changes in support systems emerged.

## Conclusion

Study findings provide useful contextual information in reaching out to the Latinx community in general—whether designing strategies to prevent or control infectious diseases, inform on chronic disease prevention, or design broad health promotion and prevention programs. Drawing on data presented here, references to science and scientists are likely to strengthen the legitimation of health messages and intervention programs aimed to reach broad Latinx communities. Study participants welcomed science-based information, whether from online sources, radio and television media, or health professionals. Though about half were not fluent in English, the absence of English proficiency is not an indicator of their level of education or even exposure to science. Most had some science education in their home countries and, hence, had a basic level of expectation for the quality of the information they received, especially if in Spanish. This may partially explain why some participants sought information from international Spanish speaking media, as shared by the woman who preferred TVE, the national television network in Spain. Second and important to health promotion and intervention programs, study participants were, to a greater or lesser extent, receptive to new information and messaging, especially if scientifically supported; and that effective communication can bring about some behavioral modification, even among those expressing strong reluctance. Such was the earlier situation when a participant expressed strong resistance to the vaccine yet became vaccinated soon after her participation in the focus group. Finally, we suggest that health messaging for Latinx populations should incorporate a multi-generational approach to deliver more expeditious sources of transmission across generations, where the flow is multidirectional across the various age strata.

Outreach messaging to these communities should be anchored by scientific support, the authenticity of the messenger, or preferably both. Focus group data presented here suggest that vaccine messaging and endorsements are best received when coming from a trained health professional or a grown child or close relative, especially when the latter are trained in the health professions. Findings presented here have the potential for designing interventions aimed at multi-origin Latinx groups to inform this population on broad themes related to health in general and focused health issues, such as vaccine development and uptake. We find that the multi-origin Latinx presence in this study facilitated a broad outreach to diverse Latinx origin groups, thus widening our exposure not only to similarities and differences, but also, and more importantly, expanding and widening our outreach to these groups.

## Data availability statement

The raw data supporting the conclusions of this article will be made available by the authors, without undue reservation.

## Ethics statement

The studies involving human participants were reviewed and approved by IRB Committee Florida International University. The patients/participants provided their written informed consent to participate in this study.

## Author contributions

All authors reviewed the study design and methods and edited the article. All authors approved this version of the article.

## Funding

Support for this study was provided by the National Institute of Health's National Institute of Minority Health and Health Disparities and National Heart, Lung, and Blood Institute (OS00000383) and National Institute of Minority Health and Health Disparities (U54MD002266) and approved by a joint IRB Florida International University and University of Miami (IRB, 20170770-MOD00052414).

## Conflict of interest

The authors declare that the research was conducted in the absence of any commercial or financial relationships that could be construed as a potential conflict of interest.

## Publisher's note

All claims expressed in this article are solely those of the authors and do not necessarily represent those of their affiliated organizations, or those of the publisher, the editors and the reviewers. Any product that may be evaluated in this article, or claim that may be made by its manufacturer, is not guaranteed or endorsed by the publisher.

## Footnotes

^1^All names for participants throughout the manuscripts are pseudonyms that have been assigned to protect participant privacy.
